# Peritoneal mesothelioma as an incidental finding during inguinal hernia surgery

**DOI:** 10.1093/jscr/rjaf1055

**Published:** 2026-01-08

**Authors:** Julian Ramin Andresen, Emerson Leonardo Monteiro, Marc Olaf Liedke

**Affiliations:** Department of Orthopaedics and Trauma Surgery, Medical University of Vienna, Währinger Gürtel 18-20, 1090 Vienna, Austria; Department of General, Visceral, and Transplant Surgery, Medical University of Graz, Auenbruggerplatz 5/5, 8036 Graz, Austria; Department of Visceral, Thoracic and Vascular Surgery, Westkuestenklinikum Heide, Academic Teaching Hospital of the Universities of Kiel, Luebeck and Hamburg, Esmarchstraße 50, 25746 Heide, Germany

**Keywords:** asbestos exposure, malignant epithelioid mesothelioma, open inguinal hernia repair, peritoneal mesothelioma, peritoneal carcinomatosis, peritoneum

## Abstract

Malignant peritoneal mesothelioma is a rare tumor disease, usually associated with asbestos exposure, which is often only diagnosed at an advanced stage. We report the case of a 52-year-old patient who was found to have marked ascites during surgery for a recurrent inguinal hernia. Subsequent diagnostics, including diagnostic laparoscopy, established the diagnosis of malignant epithelioid peritoneal mesothelioma. After tumor reduction by omentum resection and hyperthermic intraperitoneal chemotherapy, a complete remission was achieved. The case underlines the importance of differentiated clarification of unclear intra-abdominal findings in high-risk patients with possible asbestos exposure.

## Introduction

Primary malignant peritoneal mesotheliomas (MPM) are malignant neoplasms of the mesothelium of the peritoneum that can develop after exposure to asbestos. They are about 10 times rarer than pleural mesotheliomas. The incidence rate for MPM in industrialized countries is given as 0.5 to 3 per 1 000 000 cases in men and 0.2 to 2 per 1 000 000 cases in women [1]. The diagnosis is usually made at an advanced stage. In terms of differential diagnosis, MPM must be differentiated from a whole range of other neoplasms. In addition to peritoneal metastases, there are special forms such as well-differentiated papillary mesothelioma and benign multicystic mesothelioma of the peritoneum as well as adenomatoid tumors. Intraperitoneal chemotherapy is often used in combination with hyperthermia (hypothermic intra-peritoneal chemotherapy, HIPEC) and tumor resection or tumor mass reduction (cytoreductive surgery, CRS). In the case of multiple/diffuse growth of peritoneal mesothelioma, complete resection as in pleural mesothelioma is generally not possible. Mesothelioma of the peritoneum caused by asbestos is recognized as an occupational disease. Asbestos can be detected in lung tissue [2].

We report on a man who inexplicably developed increasing ascites after an inguinal hernia operation, which then resulted in a histologically confirmed malignant epithelioid mesothelioma of the peritoneum in the further diagnostic clarification.

## Case report

**Figure 1:**
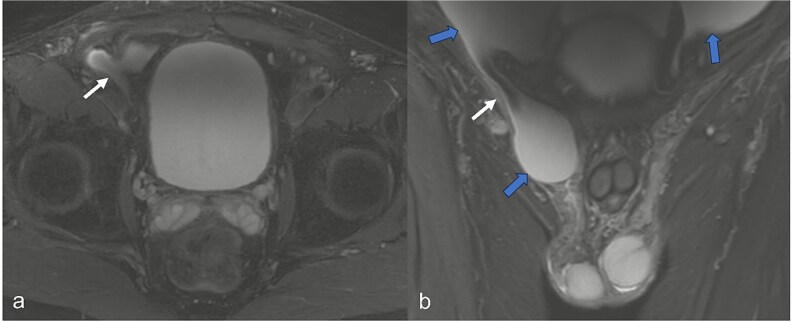
In the MRI examination of the pelvic region, the fat-suppressed, fluid-emphasized imaging in the axial section (a) shows a hernial orifice of ~1 cm (white arrow) and in the cronar section (b) the hernial orifice (white arrow) with ascites in the lesser pelvis and in the hernial sac/funiculus spermaticus (blue arrows).

**Figure 2:**
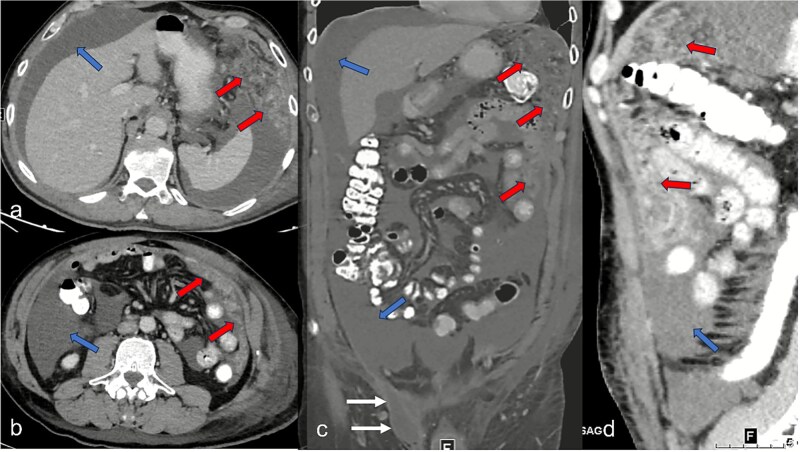
Abdominal CT imaging shows clear ascites (blue arrows) in all quadrants in the axial segments (a and b), the coronary segment (c), and sagittal segment (d), and a tumor-specific structure with thickening of the peritoneum and the greater omentum (red arrows). In (c) representation of the hernial orifice (white arrows).

**Figure 3:**
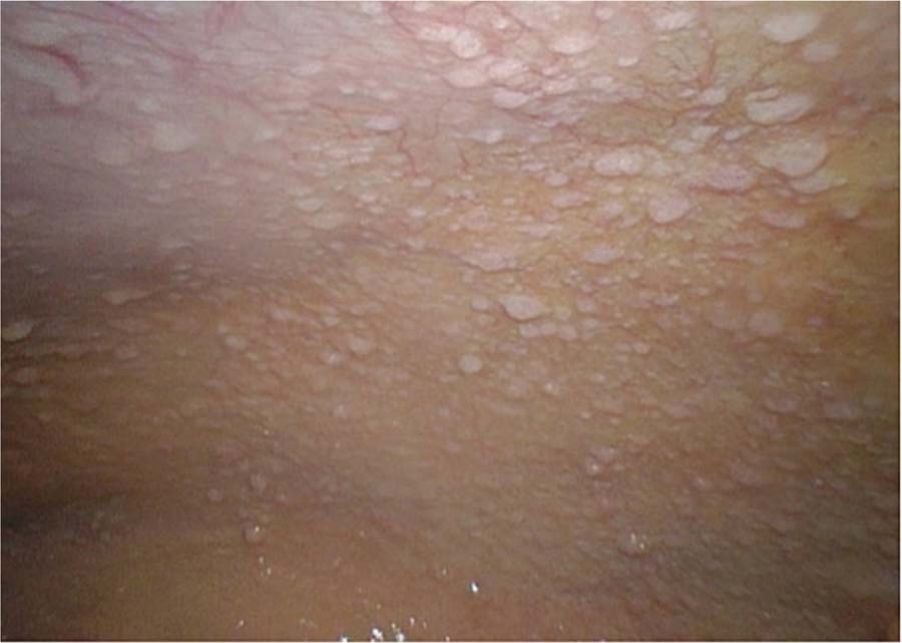
Diagnostic laparoscopy. The image shows the nodularly altered peritoneum.

**Figure 4:**
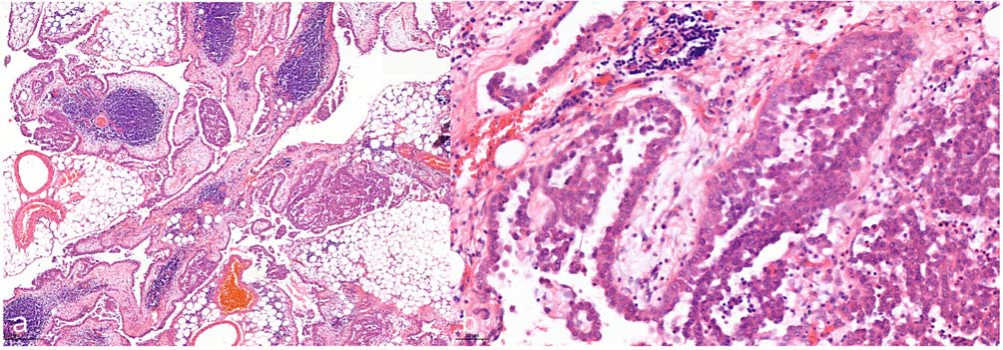
Hematoxylin and eosin staining with a 7.2-fold (a, overview image) and 40-fold (b, detail image) magnification. The histological images show a malignant epithelioid mesothelioma of the peritoneum; atypical tumor cell clusters located in papillary and exophytic formations can be seen.

A 52-year-old male patient presented for surgical treatment of a recurrent hernia in the right groin. The preoperative sonography revealed a hernial orifice with a diameter of ~1 cm. In addition, there was clear ascites with a collection of fluid in the spermatic funiculus and a suspected mural tunica vaginalis. A subsequent magnetic resonance imaging (MRI) examination of the pelvis showed a similar picture (Fig. 1). Due to the suspicious appearance of the tunica vaginalis, the recurrent hernia was not repaired at that time. In the renewed physical examination and history taking, the patient reported not only the familiar painful feeling of pressure in the area of the known inguinal hernia but also an increasing feeling of fullness over the entire abdomen. He had also lost 3 kg in weight in the last two months. He had been professionally exposed to asbestos while working as a master electrician in old houses. In the blood count, the C-reactive protein was slightly elevated at 1.88 mg/dl, the thrombocytes at 456 000/μl and the leukocytes at 12 000/μl. The subsequent computed tomography (CT) thorax and abdomen revealed a peritoneal carcinomatosis with ascites without evidence of a primary tumor (Fig. 2). An ascites puncture with subsequent cytological analysis revealed no pathological findings. The subsequent diagnostic laparoscopy revealed a four-quadrant metastasis with nodular structures on the peritoneum (Fig. 3) and the greater omentum. Biopsies taken from the peritoneum and greater omentum revealed a malignant high-grade epithelioid mesothelioma of the peritoneum (Fig. 4). After the tumor conference decision, another laparoscopy was performed to reduce the tumor tissue, after which the affected greater omentum and part of the peritoneum were removed. Hyperthermic intraperitoneal chemotherapy (HIPEC) was then carried out, which was well tolerated and led to a complete remission during the 24-month follow-up period.

An 18F-FDG PET scan and repeat abdominal CT showed no evidence of tumor recurrence, although mild ascites was still present in the pelvis. Due to persistent pressure in the area of the inguinal hernia, it was electively repaired using the Lichtenstein open technique.

## Discussion

In 94% of patients with ascites, there is no malignant etiology. An excessive amount of fluid in the abdominal cavity is a common symptom of chronic liver disease and can be found in about 3%–4% of hospitalized patients on general wards [3, 4]. Even if, as in our case, ascites cytology showed no evidence of malignancy, the rare possibility of peritoneal mesothelioma should be considered in the presence of asbestos exposure [5]. Imaging techniques, such as sonography, CT, and MRI, can describe existing pathologies and assign them to possible diseases [6, 7], but do not allow a definitive diagnosis of MPM, for which a biopsy with subsequent histological processing is necessary. The thickening of the peritoneum, the nodular structures, and the ascites described in our case are consistent with the most common histologic subtype of epithelioid mesothelioma [7]. The CT morphological description of the existing tumor mass according to location and size can be helpful for the surgical planning of tumor mass reduction. However, as in our patient, complete cytoreductive surgery is not achieved in many cases [8]. In contrast, Müller *et al.* [9] reported complete cytoreduction in 85.7% of their patients with acceptable postoperative morbidity. Acs *et al.* [10] reported a median overall survival of 38.4 months (95% CI: 23.6–54.3) and a 5-year survival rate of 42% after CRS and HIPEC. The good outcome of our patient with a recurrence-free follow-up period of 24 months is consistent with this.

There is limited experience with the treatment of recurrent hernias in patients with malignant ascites. Following extensive ascites reduction after CRS and HIPEC, elective Lichtenstein repair of the inguinal recurrent hernia was performed, which remained recurrence-free after a further 30 days.

## Conclusion

MPM can occur as a rare incidental finding during routine surgery and require consistent diagnostic clarification in the case of unclear intra-abdominal findings, particularly if there is a history of asbestos exposure. The combination of tumor reduction and HIPEC can lead to a complete remission and should be considered in good time if indicated.

To minimize recurrence, in cases of recurrent inguinal hernia with malignant ascites, elective repair should be performed after clinical improvement. Due to the prior peritonectomy and HIPEC, open inguinal hernia repair should be chosen.

## Data Availability

Data generated during and/or analyzed during the current study are available from the corresponding author on reasonable request.
